# Primary Choroid Plexus Papilloma over Sellar Region Mimicking with Craniopharyngioma: A Case Report and Literature Review

**DOI:** 10.7759/cureus.2849

**Published:** 2018-06-20

**Authors:** Chao-Hung Kuo, Yu-Shu Yen, Tsung-Hsi Tu, Jau-Ching Wu, Wen-Cheng Huang, Henrich Cheng

**Affiliations:** 1 Neurosurgery, Taipei Veterans General Hospital, Taipei, TWN

**Keywords:** sellar, choroid plexus papilloma, craniopharyngioma, endoscope, trans-sphenoidal surgery

## Abstract

Choroid plexus papillomas (CPPs) are slow-growing and benign tumors, representing less than 1% of all intracranial neoplasms. They are predominantly located in the lateral ventricles in children, and in the fourth ventricle in adults. Primary CPP over sellar regions is extremely rare. There was previously only one case reported in men.

We report the case of a 43-year-old male who initially presented with bilateral temporal hemiapnosia. A brain magnetic resonance imaging (MRI) revealed heterogenous enhanced mass lesion with cyst component compressing upward onto the optic chiasm. A craniopharyngioma was initially suspected. Further endoscopic trans-sphenoidal surgery (ETS) was performed for total tumor resection. After surgery, the patient had visual field improved without tumor recurrence on post-operative MRI in clinical follow-up. Histopathological examination of the lesion confirmed the diagnosis of CPP, with fibrovascular cores covered by a single layer of cuboidal to columnar epithelial cells.

CPP is a rare tumor without specific clinical patterns or imaging findings. Therefore, pre-operative differential diagnosis is difficult for this kind of tumor with complete separation from ventricular system. An ETS for total tumor resection provided effective treatment.

## Introduction

Choroid plexus papillomas (CPPs) are slow growing, benign tumors, representing less than 1% of all intracranial neoplasms. The tumors are most common in children, constituting 2%-4% tumors in children under 15 years of age [1]. CPPs are predominantly located in the lateral ventricles in children, and in the fourth ventricle in adults. Previous case reports described unusual CPPs arising from the pineal region [2-3], the third ventricle [4], cerebellopontine angle [5], posterior fossa [3,6], brain stem [7], suprasellar region [8], and sellar region [8-13]. To our knowledge, there was only one case report that described male patients with the primary CPPs over sellar region [13]. The general presentations of CPPs in magnetic resonance image (MRI) revealed high-intensity and isointensity mass lesion in a T1-weighted image, and isointensity and hypointensity in a T2-weighted image with T1 heterogeneous enhancement after gadolinium (Gd) injection. Here, we describe a case of CPP in the sellar region, with solid and cystic components of tumor mimicking with craniopharyngioma.

## Case presentation

**Figure 1:**
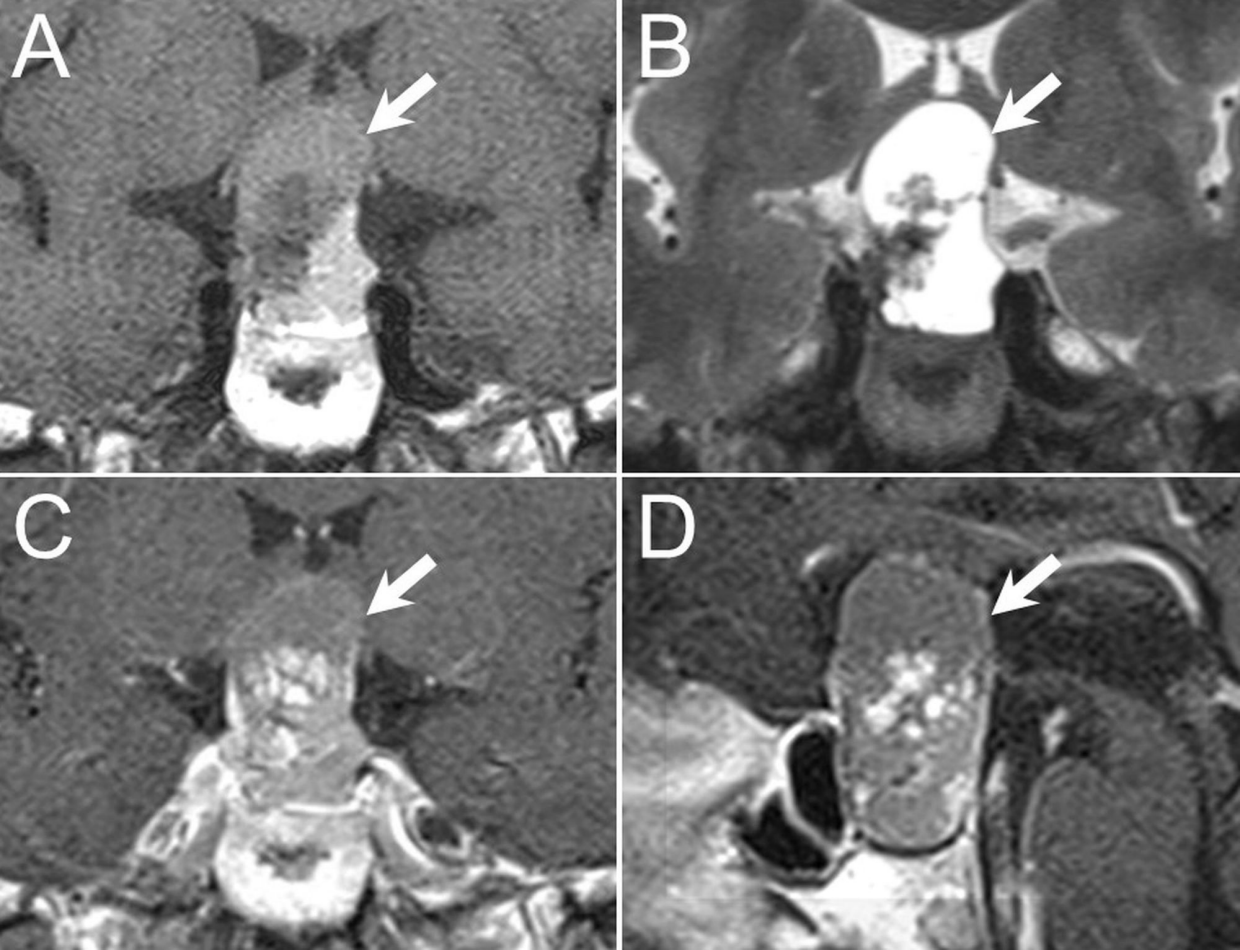
Pre-operative magnetic resonance imaging (MRI) Pre-operative MRI image revealed a mass lesion (arrows) over sellar region with upward optic nerve compression, presenting as a heterogeneous signal change over T1-weighted image (A: coronal view), and a low signal intensity in the central part surrounded peripheral high signal over T2-weighted image (B: coronal view). After gadolinium contrast injection, heterogeneous enhancement was noted over coronal (C) and sagittal (D) views.

This 43-year-old male without systemic disease complained of blurred vision progressively for almost six months. Bilateral visual field defects were found under visual field examination. The patient’s high cortical functions were intact. Cranial nerve function was also normal, except for bilateral lower temporal hemiapnosia. Anatomical MRI revealed a mass lesion over sellar and suprasellar regions with heterogeneous contrast enhancement, and a cystic component that caused upward compression of the optic chiasm (Figure 1). Endocrinological data included pre-operative serum measurements of growth hormone (GH), insulin-like growth factor-1, adrenocorticotropic hormone (ACTH), cortisol, prolactin (PRL), triiodothyronine (T3), thyroxine (T4), free T4, thyrotropin-stimulating hormone (TSH), luteinizing hormone (LH), follicle-stimulating hormone (FSH), and testosterone, all of which were at normal levels.

**Figure 2:**
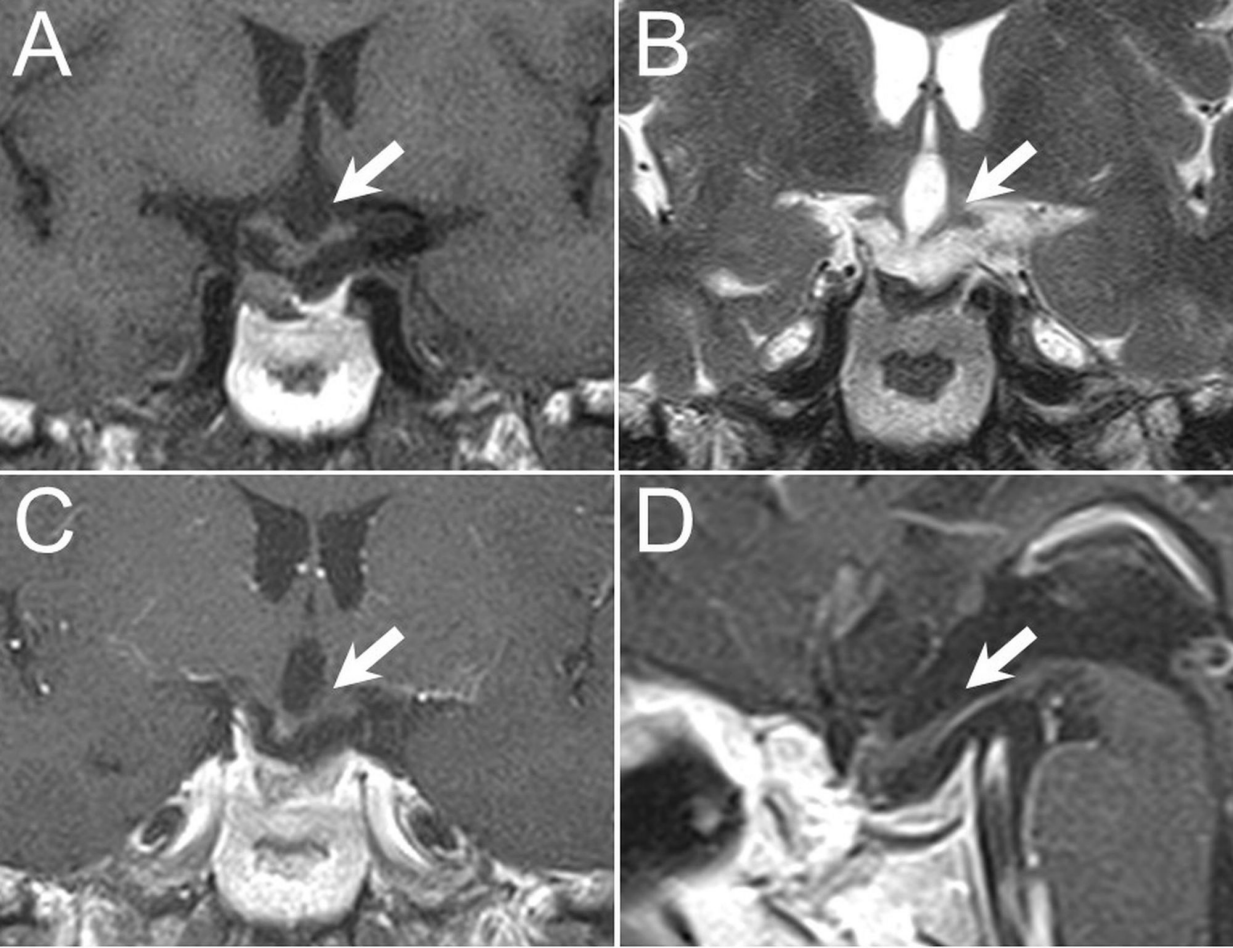
Post-operative magnetic resonance imaging (MRI) Post-operative MRI image demonstrated no residual tumor (arrows) over T1-weighted (A: coronal view), T2-weighted (B: coronal view), and gadolinium-enhanced T1-weighted images (C: coronal view, D: sagittal view).

After the provisional diagnosis of craniopharyngioma with optic nerve compression, we performed endoscopic trans-sphenoidal surgery (ETS) for tumor removal. We used a standard one-surgeon, two-hand technique via a single nostril, with the endoscope mounted on a pneumatic scope holder. A vertical linear mucosal incision was made with electric cautery near the root of the bony nasal septum. A nasal speculum was placed after dilatation of the space and fracture of the bony nasal septum without destruction of the middle turbinate. Subsequently, posterior septectomy was performed with removal of the anterior portion of the vomer bone using high-speed drills and Kerrison rongeurs. The bony septum inside the sphenoid sinus and the rostrum of sphenoid bone were removed to expose the sella turcica. The floor of the pituitary fossa was removed after confirmation of anatomy via the intraoperative navigation system or lateral skull fluoroscopy. A soft density tumor with some yellowish particles was removed endoscopically with ring curettes over the sellar region. The cystic part of the tumor with old blood content was drained during surgery. The tumor was completely removed with the diaphragm sella intact and without cerebrospinal fluid leakage. The sellar floor was reconstructed using with tissue glue and autologous bone grafts harvested during the approach. The patient had no neurological deficit after surgery. No electrolyte imbalance, diabetes insipitus (DI) sign, or hormone deficiency was noted. The original visual field defect also improved after surgery. Post-operative images indicated no residual or recurrent tumor within the two-year follow-up period (Figure 2).

**Figure 3:**
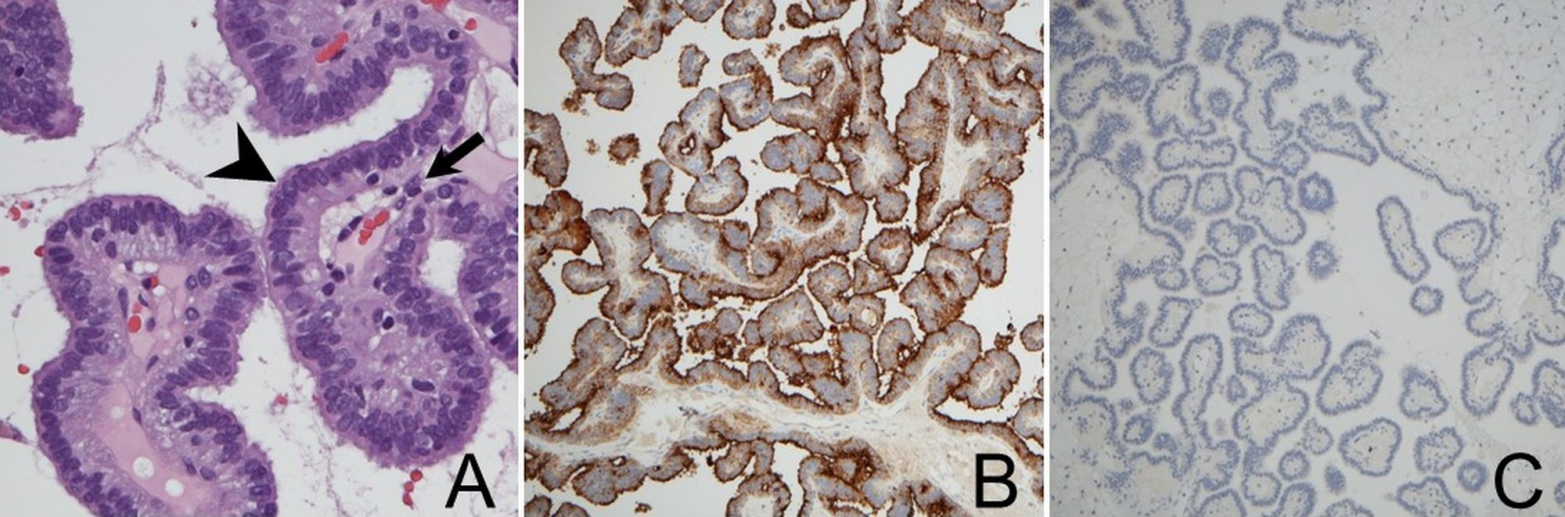
Pathology A: Photographs of the surgical specimen illustrating a papillary structure with a layer of columnar epithelial cells (arrowhead) and fibrovascular stroma (arrow, hematoxylin and eosin stain, 100x magnification). B: The tumor cells were positive for epithelial membrane antigen (100x magnification). C: The tumor cells were negative for glial fibrillary acidic protein (100x magnification).

Microscopic examination revealed that the tumor was composed of delicate fibrovascular cores covered by a single layer of cuboidal to columnar epithelial cells (Figure 3A). There are no anaplastic features identified. The tumor cells were immunoreactive for cytokeratin AE1/AE3 and epithelial membrane antigen (EMA), focally positive for S-100, and non-reactive for glial fibrillary acidic protein (GFAP) (Figures 3B-3C). Immunohistochemical stain with multiple pituitary hormones, including ACTH, PRL, FSH, LH, GH, and TSH, was negative. Based on these results, we diagnosed CPP.

## Discussion

The first case of primary CPP over the sellar region in a female patient was presented in 1992 [8]. Including our case, there have been seven published cases in the literature (Table 1) [8-13]. These lesions were all located over the sella turcica with suprasellar extension. Two cases had hormone dysfunction with higher prolactin or/and lower T4 level [9,12] and one of them had clinical presentations of amenorrhea and galactorrhea [9]. Visual field defect and headache were the most common clinical presentations for the patients with CCPs over the sellar region. In the published literature, the sellar lesion revealing homogenous enhancement with or without cystic component was the common presentation in MRI examinations. However, in our case, atypical presentation of the sellar lesion with heterogenous contrast enhancement, mimicking with craniopharyngioma, was demonstrated.

**Table 1:** Summary of primary choroid plexus papilloma (CPP) over sellar region ETS: endosopic trans-sphenoidal surgery.

Author	Age/sex	Symptoms	Location	Hormone	Treatment	Outcome
Kimura et al. [8]	34/female	Visual filed defect, left side	Sella, suprasellar	No described	Craniotomy	No described
Ma et al. [10]	49/female	Visual field defect, left side	Sella, suprasellar	Normal	ETS †	Left side vision improved
Sameshima et al. [11]	51/female	Headache	Sella, suprasellar	Normal	ETS †	No described
Bian et al. [9]	31/female	Amenorrhea and galactorrhea	Sella, suprasellar	Higher prolactin level	ETS†	Recovered with regular menses cycle
Keskin et al. [13]	50/male	Visual filed defect, left side	Sella, suprasellar	Normal	Craniotomy	Left side vision improved
Gong et al. [12]	43/female	Headache, visual defect, left side	Sella, suprasellar	Lower free T4 and higher prolactin levels	ETS†	Recurrence, re-operation (craniotomy)
Our case	43/male	Bilateral hemiapnosia	Sella, suprasellar	Normal	ETS†	Bilateral vision improved

Neuroimaging presentation of craniopharyngioma, our initial diagnosis, is not specific. Craniopharyngioma may be hyperintense, iso-intense, or hypointense on a T1-weighted image, and hyperintense on a T2-weighted image. The high signal intensity of cystic fluid on a T1-weighted image may be caused by high protein concentrations. Cholesterol and triglycerides do not affect the signal intensity on a T1-weighted image, but may cause high signal change on a T2-weighted image [14]. Based on imaging alone, our case resembled these criteria. Therefore, our pre-operative diagnosis was craniopharyngioma. Based on image and tumor location alone, it is difficult to diagnose CPP before surgery. A post-operative pathological examination is the only way to make a definitive diagnosis.

Complete surgical tumor resection is recommended for treatment of CPP [15-16]. An ETS provided a dynamic and magnified inspection of the areas of tumor resection. It also caused a smaller cosmetic effect compared to craniotomy. Most of the previous cases had clinical improvement after surgery. Gong et al. [12] illustrated the only one case of CPP with recurrence; a 43-year-old female with a pigmented CPP underwent the primary single-nostril ETS. A craniotomy with a subfrontal approach was performed three months after the ETS because of tumor recurrence and was followed by adjunctive radiotherapy. However, there was no strong or enough evidence that adjuvant treatment was needed after the primary surgery.

## Conclusions

CPP is a rare tumor without specific clinical patterns on image findings. Primary CPP over the sellar region is extremely rare, especially in men. A pre-operative differential diagnosis is difficult for this type of tumor with complete separation from ventricular system. ETS for complete tumor resection provided an effective treatment.

## References

[1] Gjerris F, Agerlin N, Borgesen SE (1998). Epidemiology and prognosis in children treated for intracranial tumours in Denmark 1960-1984. Childs Nerv Syst.

[2] Kawahara I, Tokunaga Y, Yagi N, Iseki M, Abe K, Hayashi T (2007). Papillary tumor of the pineal region. Neurol Med Chir (Tokyo).

[3] Noguchi A, Shiokawa Y, Kobayashi K (2004). Choroid plexus papilloma of the third ventricle in the fetus. Case illustration. J Neurosurg.

[4] Ni HC, Piao YS, Lu DH, Fu YJ, Ma XL, Zhang XJ (2013). Chordoid glioma of the third ventricle: four cases including one case with papillary features. Neuropathology.

[5] Casar Borota O, Jacobsen EA, Scheie D (2006). Bilateral atypical choroid plexus papillomas in cerebellopontine angles mimicking neurofibromatosis 2. Acta Neuropathol.

[6] Beskonakli E, Cayli S, Bostanci U, Kulacoglu S, Yalcinlar Y (1998). Choroid plexus papillomas of the posterior fossa: extraventricular extension, intraventricular and primary extraventricular location. Report of four cases. J Neurosurg Sci.

[7] Pillai A, Rajeev K, Chandi S, Unnikrishnan M (2004). Intrinsic brainstem choroid plexus papilloma. Case report. J Neurosurg.

[8] Kimura M, Takayasu M, Suzuki Y (1992). Primary choroid plexus papilloma located in the suprasellar region: case report. Neurosurgery.

[9] Bian LG, Sun QF, Wu HC, Jiang H, Sun YH, Shen JK (2011). Primary choroid plexus papilloma in the pituitary fossa: case report and literature review. Acta Neurochir (Wien).

[10] Ma YH, Ye K, Zhan RY, Wang LJ (2008). Primary choroid plexus papilloma of the sellar region. J Neurooncol.

[11] Sameshima T, Tanikawa R, Sugimura T (2010). Choroid plexus papilloma originating in the sella turcica--case report. Neurol Med Chir (Tokyo).

[12] Gong X, Liu C, Zhang L, Li Z, Bartley CM, Liu Z (2017). A primary pigmented choroid plexus papilloma located within the sella turcica: case report and literature review. World Neurosurg.

[13] Keskin F, Erdi F, Kaya B, Toy H (2016). Sellar-suprasellar extraventricular choroid plexus papilloma: a case report and review of the literature. J Korean Neurosurg Soc.

[14] Shin JL, Asa SL, Woodhouse LJ, Smyth HS, Ezzat S (1999). Cystic lesions of the pituitary: clinicopathological features distinguishing craniopharyngioma, Rathke's cleft cyst, and arachnoid cyst. J Clin Endocrinol Metab.

[15] Boyd MC, Steinbok P (1987). Choroid plexus tumors: problems in diagnosis and management. J Neurosurg.

[16] McGirr SJ, Ebersold MJ, Scheithauer BW, Quast LM, Shaw EG (1988). Choroid plexus papillomas: long-term follow-up results in a surgically treated series. J Neurosurg.

